# *C9orf72*-derived arginine-containing dipeptide repeats associate with axonal transport machinery and impede microtubule-based motility

**DOI:** 10.1126/sciadv.abg3013

**Published:** 2021-04-09

**Authors:** Laura Fumagalli, Florence L. Young, Steven Boeynaems, Mathias De Decker, Arpan R. Mehta, Ann Swijsen, Raheem Fazal, Wenting Guo, Matthieu Moisse, Jimmy Beckers, Lieselot Dedeene, Bhuvaneish T. Selvaraj, Tijs Vandoorne, Vanesa Madan, Marka van Blitterswijk, Denitza Raitcheva, Alexander McCampbell, Koen Poesen, Aaron D. Gitler, Philipp Koch, Pieter Vanden Berghe, Dietmar Rudolf Thal, Catherine Verfaillie, Siddharthan Chandran, Ludo Van Den Bosch, Simon L. Bullock, Philip Van Damme

**Affiliations:** 1 KU Leuven—University of Leuven, Department of Neurosciences, Experimental Neurology and Leuven Brain Institute (LBI), Leuven, Belgium.; 2 VIB, Center for Brain & Disease Research, Laboratory of Neurobiology, Leuven, Belgium.; 3 Division of Cell Biology, MRC Laboratory of Molecular Biology, Cambridge, UK.; 4 Department of Genetics, Stanford University School of Medicine, Stanford, CA, USA.; 5 UK Dementia Research Institute, University of Edinburgh, Edinburgh, UK.; 6 Centre for Clinical Brain Sciences, University of Edinburgh, Edinburgh, UK.; 7 The Anne Rowling Regenerative Neurology Clinic, University of Edinburgh, Edinburgh, UK.; 8 The Euan MacDonald Centre, University of Edinburgh, Edinburgh, UK.; 9 KU Leuven—University of Leuven, Department of Development and Regeneration, Stem Cell Institute, Leuven, Belgium.; 10 KU Leuven—University of Leuven, Department of Neurosciences, Laboratory for Molecular Neurobiomarker Research and Leuven Brain Institute (LBI), Leuven, Belgium.; 11 KU Leuven—University of Leuven, Department of Imaging and Pathology, Laboratory for Neuropathology and Leuven Brain Institute (LBI), Leuven, Belgium.; 12 Laboratory Medicine, University Hospitals Leuven, Leuven, Belgium.; 13 Department of Neuroscience, Mayo Clinic, Jacksonville, FL, USA.; 14 Biogen Idec, Boston, MA, USA.; 15 Hector Institute for Translational Brain Research, Central Institute of Mental Health, University of Heidelberg, Heidelberg, Germany.; 16 Institute of Reconstructive Neurobiology, Life & Brain Center, University of Bonn, Bonn, Germany.; 17 KU Leuven—University of Leuven, Translational Research Centre for Gastrointestinal Disorders, Leuven, Belgium.; 18 Department of Pathology, University Hospitals Leuven, Leuven, Belgium.; 19 Centre for Brain Development and Repair, inStem, Bangalore, India.; 20 MRC Centre for Regenerative Medicine, University of Edinburgh, Edinburgh, UK.; 21 Department of Neurology, University Hospitals Leuven, Leuven, Belgium.

## Abstract

A hexanucleotide repeat expansion in the *C9orf72* gene is the most common genetic cause of amyotrophic lateral sclerosis (ALS) and frontotemporal dementia (FTD). How this mutation leads to these neurodegenerative diseases remains unclear. Here, we show using patient stem cell–derived motor neurons that the repeat expansion impairs microtubule-based transport, a process critical for neuronal survival. Cargo transport defects are recapitulated by treating neurons from healthy individuals with proline-arginine and glycine-arginine dipeptide repeats (DPRs) produced from the repeat expansion. Both arginine-rich DPRs similarly inhibit axonal trafficking in adult *Drosophila* neurons in vivo. Physical interaction studies demonstrate that arginine-rich DPRs associate with motor complexes and the unstructured tubulin tails of microtubules. Single-molecule imaging reveals that microtubule-bound arginine-rich DPRs directly impede translocation of purified dynein and kinesin-1 motor complexes. Collectively, our study implicates inhibitory interactions of arginine-rich DPRs with axonal transport machinery in *C9orf72*-associated ALS/FTD and thereby points to potential therapeutic strategies.

## Teaser

Arginine-rich dipeptide repeats associated with ALS and FTD inhibit machinery for microtubule-based axonal cargo transport.

## INTRODUCTION

Amyotrophic lateral sclerosis (ALS) and frontotemporal dementia (FTD) are adult-onset neurodegenerative diseases that are characterized by the degeneration of motor neurons in the spinal cord, brainstem and motor cortex, and neurons in the frontal and anterior temporal cortex, respectively (*1*, *2*). ALS patients present with progressive muscle weakness and wasting, whereas FTD patients present with behavioral and/or language abnormalities (*1*, *2*). In recent years, it has become evident that ALS and FTD belong to a spectrum of disorders, sharing some clinical, neuropathological, and genetic features (*1*, *2*). A GGGGCC (G_4_C_2_) repeat expansion in the 5′ noncoding region of the *C9orf72* gene is the most common genetic cause of both ALS and FTD (*C9*-ALS/FTD) (*3*, *4*). Three non-mutually exclusive pathological mechanisms have been proposed for the hexanucleotide repeat expansions (HREs) (*5*). The first is a loss-of-function scenario due to decreased expression of *C9orf72* mRNA and protein observed in *C9*-ALS/FTD patients. The second is an RNA gain-of-function mechanism caused by the accumulation of expanded repeat transcripts that sequester numerous RNA-binding proteins. The third is a protein gain of function via the generation of dipeptide repeat (DPR) proteins originating from non-ATG–mediated translation of the expanded repeat transcripts.

This repeat-associated non-ATG (RAN) translation occurs in all reading frames of sense and antisense transcripts, resulting in five DPR proteins: poly-glycine-arginine (GR) and poly-glycine-alanine (GA) exclusively from the sense transcript, poly-proline-arginine (PR) and poly-proline-alanine (PA) exclusively from the antisense transcript, and poly-glycine-proline (GP) from both transcripts (*5*). DPRs are found in cytoplasmic inclusions in *C9*-ALS/FTD postmortem brain and spinal cord tissue and are also detected in motor neurons differentiated from patient-derived induced pluripotent stem cells (iPSCs) (*5*, *6*). The arginine-rich DPRs—poly-PR and poly-GR—are potently toxic in numerous disease models (*7*–*16*) and cause mitochondrial (*17*, *18*) and endoplasmic reticulum (ER) stress (*15*), as well as disturbances in gene expression, RNA processing and translation (*7*, *19*–*23*), nucleocytoplasmic transport (*10*, *11*, *14*, *24*, *25*), and the dynamics of membrane-less organelles (*26*–*28*). Whether these effects fully explain the toxicity of arginine-rich DPRs in neurons remains unclear.

Of the several other genes that have been associated with ALS (*29*), three encode proteins important for microtubule-based cargo transport: the tubulin isotype α4a (*30*), the plus end-directed kinesin-1 motor KIF5A (*31*), and DCTN1, a component of the dynactin complex that activates the minus end-directed motor cytoplasmic dynein-1 (hereafter dynein) (*32*). The association of these mutations with ALS suggests that motor neurons, which are selectively targeted by the disease, are particularly reliant on efficient cargo trafficking, presumably due to their extended processes. This notion prompted us to investigate the involvement of microtubule-based transport in *C9*-ALS/FTD.

We observe impaired microtubule-based transport in iPSC-derived motor neurons from *C9*-ALS/FTD patients, including elevated pausing of motile cargoes. We show that exposure to poly-PR and poly-GR elicits comparable effects in control iPSC-derived motor neurons and *Drosophila* neurons within the intact animal. Interaction studies, including in motor neurons and postmortem patient tissues, reveal association of arginine-rich DPRs with microtubules and motor proteins. We use single-molecule imaging of purified components to demonstrate that the arginine-rich DPRs directly impede motility by binding both the unstructured tubulin tails of microtubules and dynein and kinesin-1 motor complexes. Our data strengthen the evidence that defective axonal cargo trafficking contributes to ALS pathogenesis and implicate inhibitory interactions of arginine-rich DPRs with the axonal transport machinery in *C9*-ALS/FTD.

## RESULTS

### Characterization of *C9*-ALS/FTD motor neurons

To investigate whether *C9*-ALS/FTD is associated with impaired microtubule-based transport, we first generated spinal motor neurons (sMNs) from fibroblast-derived iPSC lines from four *C9orf72* patients and three healthy controls (figs. S1 to S3 and table S1). Molecular analysis of *C9orf72* cell lines revealed expansions of ~180 to 770 hexanucleotide repeats (fig. S2B). With the exception of a slightly elevated rate of neurite outgrowth (fig. S3F), *C9orf72* neurons developed indistinguishably to the controls. Immunostaining showed that the *C9orf72* lines produced motor neurons with similar efficiency to control lines at day 38 of the in vitro differentiation (fig. S3). Whole-cell patch clamp recordings of evoked and spontaneous action potentials at the same time point demonstrated functional maturation of both control and *C9orf72* sMN lines, with no differences in electrical activity between the genotypes (fig. S4 and table S2). This finding is in line with previous studies using iPSC-derived sMNs (*33*, *34*).

We next investigated potential loss-of-function mechanisms in the sMNs by assessing *C9orf72* mRNA levels using digital droplet quantitative reverse transcription polymerase chain reaction (qRT-PCR). Although there were some subtle differences in the relative levels of specific *C9orf72* transcript variants between patient and control sMNs, there were no differences in overall *C9orf72* mRNA levels (fig. S5, A to D). Immunoblotting showed that levels of C9orf72 protein were also not significantly different between patient-derived lines and controls (fig. S5, E and F). We did, however, detect both poly-GA and poly-GP specifically in the *C9orf72* lines using an enzyme-linked immunosorbent assay (ELISA) (fig. S5G), confirming that RAN translation is occurring in these cells.

### *C9orf72* hexanucleotide expansions impair microtubule-based transport in motor neurons

**Fig. 1:**
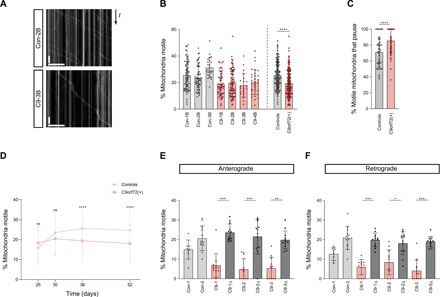
The *C9orf72* HRE impairs mitochondrial transport in sMN neurites. (**A**) Example kymographs (time-distance plots) of mitochondria (MitoTracker Red) from 38-day-old control (CON) and *C9orf72* (C9) sMNs. Scale bars, 50 s and 30 μm. (**B**) Percentage of mitochondria that are motile in sMNs (day 38). (**C**) Percentage of all motile mitochondria that undergo pausing (day 38). (**D**) Percentage of all mitochondria that are motile at different days of differentiation [day 38 data reproduced from (B)]. (**E** and **F**) Percentage of mitochondria (mitoDsRed2) transported anterogradely (E) or retrogradely (F) in 38-day-old *C9orf72* sMNs or isogenic paired controls with excision of the HRE. Two independent healthy controls are shown for comparison. Difference in magnitude of transport deficits compared to (B) may be related to different mitochondrial labeling methods. Data represented as means ± SD; dots represent values for individual neurites (see table S3 for numbers of neurites analyzed). Statistical significance was evaluated with a Mann-Whitney *U* test (B to D) or a Kruskal-Wallis test with Dunn’s multiple comparison (E and F) (*P < 0.05; **P < 0.01; ***P < 0.001; ****P < 0.0001; ns, not significant; three independent differentiations).

Defective microtubule-based transport of mitochondria in axons has been linked with a variety of neurodegenerative diseases, including ALS caused by other mutations (*35*). We therefore assessed motility of these organelles by live imaging of *C9orf72* and healthy control motor neuron lines incubated with the mitochondrial stain MitoTracker. Kymograph-based analysis of neurons that had been differentiated for 38 days revealed that the proportion of mitochondria undergoing transport in neurites was reduced in the *C9orf72* lines (Fig. 1, A and B, and fig. S6, A to D). The total number of mitochondria in neurites was also lower in *C9orf72* lines (fig. S6D), a phenomenon previously observed when microtubule motors are inhibited in neurons due to a deficit in motor-driven export of these organelles from the soma (*36*, *37*). Furthermore, a greater proportion of the mitochondria that were motile in neurites paused during or at the end of processive movements in the *C9orf72* lines compared to the control lines (Fig. 1C). We also observed impaired transport of RNA granules [which were labeled with the SYTO-RNA Select dye (*38*)] in the *C9orf72* sMNs at 38 days of differentiation (fig. S7). We next investigated the time course of transport deficits in the patient-derived neurons. Impaired motility of MitoTracker-stained mitochondria developed over time in culture (not evident at days 25 and 30) and persisted in older *C9orf72* neurons (day 52) (Fig. 1D and fig. S8). Collectively, these data reveal an age-related onset of transport deficits in the patient-derived neurons.

The dense nature of the neuronal cultures meant that we were unable to assign anterograde and retrograde movements when mitochondria in all neurons were labeled with MitoTracker. We therefore visualized mitochondria in a subset of neurons using sparse infection with lentivirus expressing mitoDsRed2. This analysis showed that both anterograde and retrograde motion was impaired in *C9orf72* neurons (Fig. 1, E and F). These experiments were performed in independent *C9orf72* iPSC lines using isogenic controls in which the repeat expansion had been corrected by CRISPR/Cas9 gene editing (*33*). We conclude from these observations that the *C9orf72* repeat expansion impairs both anterograde and retrograde motion.

### Arginine-rich DPRs disrupt microtubule-based transport in sMNs

**Fig. 2:**
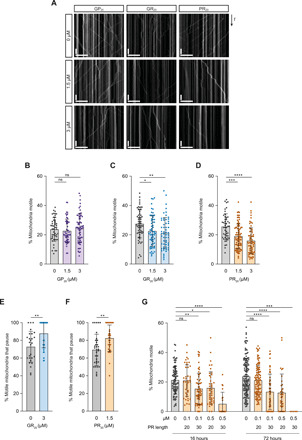
Arginine-rich DPRs impair mitochondrial transport in sMNs of healthy individuals. (**A**) Example kymographs after treatment of control neuron with synthetic GP_20_, GR_20_, or PR_20_ (1.5 and 3 μM). Scale bars, 50 s and 30 μm. (**B** to **D**) Percentage of all mitochondria that are motile upon treatment with GP_20_, GR_20_, or PR_20_. (**E** and **F**) Percentage of all motile mitochondria that undergo pausing after treatment with GR_20_ or PR_20_. (**G**) Percentage of all mitochondria that are motile after 16- or 72-hour treatment with PR_20_ or PR_30_ (0.1 or 0.5 μM). Data represented as means ± SD; dots represent values for individual neurites (see table S3 for numbers of neurites analyzed). Statistical significance was evaluated with a Kruskal-Wallis test with Dunn’s multiple comparison (B to D and G) or a Mann-Whitney *U* test (E and F) [*P < 0.05; **P < 0.01; ***P < 0.001; ****P < 0.0001; (B to D) three independent experiments; (E to G) two independent experiments except for data from 72-hour PR_20_ treatment (*N* = 4)].

As introduced above, there is compelling evidence that arginine-rich DPRs produced by *C9orf72* HREs contribute to pathophysiology (*7*–*15*, *18*, *24*, *25*). We therefore tested whether these molecules are sufficient to recapitulate the transport deficits observed in *C9orf72* patient–derived motor neurons. We added peptides containing 20 repeats of PR or GR (PR_20_ and GR_20_) to the culture medium of control iPSC-derived sMNs. Consistent with previous observations (*8*, *15*), these peptides were taken up by the neurons (fig. S9, A to C). We observed a reduced frequency of mitochondrial transport in neurons upon overnight treatment with arginine-rich DPRs at concentrations that did not trigger cell death (Fig. 2, A, C, and D, and figs. S9D and S10, D to I). As was observed in *C9orf72* neurons, sMNs treated with arginine-rich DPRs also had a reduction in the number of mitochondria in neurites (fig. S10, F and I). Treated neurons had a greater proportion of mitochondria that arrested during or at the end of a processive movement (Fig. 2, E and F), which was also a feature of the *C9orf72* lines (Fig. 1C).

To investigate the effect of repeat length on transport, we treated sMNs with PR_30_. Dose-response experiments revealed that PR_30_ was more potent than PR_20_ in our assay, significantly reducing mitochondrial motility upon overnight treatment with a concentration in the medium as low as 0.1 μM (Fig. 2G and figs. S11 and S12). We also evaluated the effect of a prolonged exposure of peptides on transport, finding that 3 days of incubation with poly-GR or poly-PR also impaired transport in a dose-dependent manner (Fig. 2G and figs. S11 and S13). Whereas overnight treatment with PR_30_ partially inhibited motility, 3 days of incubation with this peptide prevented all transport, indicating stronger inhibition with increasing exposure time. Delivery into neurons of poly-GP (GP_20_), which was nontoxic in other studies (*10*, *26*, *27*) and in this one (fig. S9D), did not affect motility of mitochondria (Fig. 2, A and B, and figs. S10, A to C, and S13, E to H). As was observed in untreated *C9orf72* lines, control motor neurons treated with arginine-rich DPRs had impaired transport of RNA granules (fig. S14, A and F to O). This effect was not observed with poly-GP (fig. S14, A to E). Collectively, these observations show that arginine-rich DPRs inhibit transport in human sMNs and that the effects on motility are similar to those observed in *C9orf72* patient–derived neurons.

### Microtubule-based transport in adult *Drosophila* is impaired by arginine-rich DPRs

**Fig. 3:**
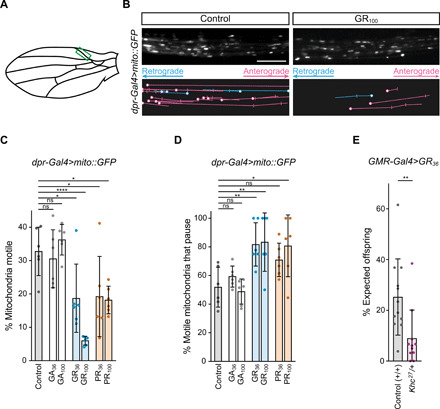
Mitochondrial transport in adult *Drosophila* axons is impaired by arginine-rich DPRs. (**A**) Cartoon of *Drosophila* wing. Green box, imaged region containing bundled axons from multiple neurons. (**B** to **D**) Effects of arginine-rich DPRs on transport of mitochondria (mito::GFP) in 2-day-old males (mito::GFP and peptides were expressed specifically in chemosensory neurons with *dpr-Gal4*). Control, no DPR expression. (B) Example stills of mitochondria and traces of transport events in corresponding movies. Blue, retrograde tracks; magenta, anterograde tracks; circles, starting locations; vertical lines, pausing sites. Scale bar, 10 μm; movie duration, 3 min. (C) Percentage of all mitochondria that are motile. (D) Percentage of all motile mitochondria that undergo pausing. (**E**) Effect of *Kinesin-1 heavy chain* (*Khc*) gene dosage on GR_36_-induced lethality (scored 4 to 6 days after eclosion). Data expressed as percentage of offspring expected when there is no lethality. *Khc^27^*, kinesin-1 null allele. In (C) to (E), means ± SD are shown; dots represent values for individual wings (C and D) or individual egg lays (E) (see table S4 for numbers analyzed). Statistical significance was evaluated with a one-way analysis of variance (ANOVA) with Dunnett’s multiple comparisons correction (C and D) or a Mann-Whitney *U* test (E) (*P < 0.05; **P < 0.01; ****P < 0.0001).

We next explored the effect of poly-GR and poly-PR on axonal transport in vivo. We used a previously described adult *Drosophila* model, in which microtubule-based movements of green fluorescent protein (GFP)–labeled mitochondria are visualized in wing nerve axons of immobilized flies (Fig. 3, A and B) (*36*). Here, as in other axons (*37*, *39*), anterograde and retrograde mitochondrial movements are driven by kinesin-1 and dynein motors, respectively (*36*). There was a decrease in the frequency of axonal transport of mitochondria upon transgenic expression of poly-PR and poly-GR in wing neurons (Fig. 3, B and C). This effect was evident in both the anterograde and retrograde directions (fig. S15, A and B). Arginine-rich DPRs also increased the incidence of mitochondria pausing within or at the end of a processive movement (Fig. 3, B and D), as was observed in human sMNs treated with these peptides or derived from *C9*-ALS/FTD patients (Figs. 1C and 2, E and F). Poly-GA, which is relatively highly expressed from *C9orf72* repeat expansions (*40*), did not affect mitochondrial trafficking when expressed in the wing nerve (Fig. 3, C and D). These observations show that arginine-rich DPRs are sufficient to impair bidirectional cargo transport in vivo.

We next used *Drosophila* to investigate whether transport deficits contribute to the cellular toxicity of arginine-rich DPRs. We took previously established models in which poly-GR and poly-PR are expressed in other tissues of the fly (*9*) and asked if the phenotypes are exacerbated by reducing motor gene dosage (Fig. 3E, fig. S16, and table S4). Inactivating one copy of the *Khc* gene, which encodes the kinesin-1 heavy chain, strongly enhanced the lethality (Fig. 3E and table S4) and eye defects (fig. S16) associated with expression of these DPRs. Control experiments indicated that the modification of arginine-rich DPR toxicity was due to the mutant *Khc* allele and not another mutation in the genetic background (fig. S16). The enhancement of the poly-GR and poly-PR phenotypes by reducing *Khc* gene dosage is consistent with the contribution of transport defects to the toxicity of the peptides.

### Arginine-rich DPRs interact with microtubules and motor proteins

**Fig. 4:**
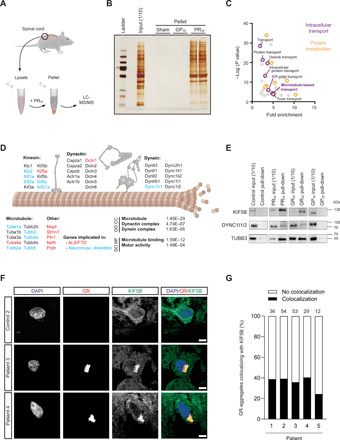
Arginine-rich DPRs interact with axonal transport machinery. (**A**) Overview of mass spectrometry experiment. (**B**) Silver stain of pellet fractions. PR_30_, but not GP_30_, induces protein phase separation. (**C**) The PR_30_ interactome (*n* = 1811) is enriched for GO categories centered on protein metabolism (including factors involved in translation, folding, and degradation) and intracellular transport. (**D**) Illustration of components of axonal transport machinery present in PR_30_ interactome (GO CC, GO analysis cellular compartment; GO MF, GO analysis molecular function). Proteins associated with ALS/FTD or other neuromuscular disorders are highlighted in red and blue, respectively. (**E**) Immunoblots confirming interactions of KIF5B, dynein, and tubulin with PR_20_ and GR_20_, but not GP_20_, in human sMN lysates (uncropped blots shown in fig. S17, C to E). In (B) and (E), the input was diluted 1:10 before gel loading. (**F**) Immunofluorescence of frontal cortex from *C9orf72* patients showing examples of enrichment of KIF5B in poly-GR aggregates. ALS patients without a *C9orf72* expansion were used as controls. Scale bar, 5 μm. (**G**) Quantification of colocalization of poly-GR inclusions and KIF5B foci (number of poly-GR inclusions analyzed for each patient is shown above the bar).

Arginine-rich DPRs might interfere with the transport machinery directly or by perturbing another cellular process that is indirectly required for efficient trafficking. To determine whether direct binding of arginine-rich DPRs to the microtubule-based transport machinery could be behind the axonal transport defects, we first asked if PR_30_ interacts with tubulin or microtubule motors in mouse spinal cord lysate. As previously observed with cancer cell extracts (*26*), incubating the soluble fraction of the spinal cord lysate with PR_30_ peptide, but not GP_30_, led to the formation of large biomolecular condensates that could be pelleted by gentle centrifugation and analyzed by mass spectrometry (Fig. 4, A to C). The spinal cord interactome of PR_30_ was strongly enriched for gene ontology (GO) categories (e.g., RNA granule, translation, proteasome, and protein folding) present in the cancer cell interactome (Fig. 4C; fig. S17, A and B; and table S5) (*26*). We also identified a strong enrichment of terms related to intracellular transport (Fig. 4, C and D). In addition to factors involved in nucleocytoplasmic transport, a process we and others previously implicated in *C9*-ALS/FTD (*10*, *11*, *14*, *24*), this category included many proteins involved in microtubule-based transport (Fig. 4C and table S5). Several isotypes of the α- and β-tubulin subunits of microtubules were precipitated with PR_30_ (Fig. 4D and table S5), as were the three different kinesin-1 isoforms (Kif5A, Kif5B, and Kif5C) and components of the dynein and dynactin complexes (Fig. 4D and table S5). We also detected other classes of kinesins and components of intraflagellar transport dynein (cytoplasmic dynein-2) in the precipitate (Fig. 4D and table S5). These data reveal that PR_30_ can associate in spinal cord lysate with multiple proteins involved in microtubule-based transport (Fig. 4D and table S5). Several of these factors are mutated in ALS or other neuromuscular disorders (Fig. 4D, fig. S17A, and table S5).

The association of arginine-rich DPRs with kinesin-1, dynein, and microtubules was further supported by immunoprecipitations from human iPSC-derived sMNs treated with hemagglutinin (HA)–tagged PR_20_, GR_20_, and GP_20_ peptides (Fig. 4E and fig. S17, C to E). In these experiments, robust signals for KIF5B, tubulin, and dynein were observed in immunoblots of the material precipitated with PR_20_ and GR_20_, but not in the material precipitated with GP_20_.

We next investigated interactions of DPRs with microtubules and motors within intact sMNs using fluorescence microscopy. The broad distribution of microtubules, KIF5B, and dynein within cell bodies and along neuronal processes prevented meaningful analysis of interactions with exogenous DPRs at these sites by conventional colocalization analysis. However, colocalization of signals from poly-PR and tubulin could be detected in growth cones of developing sMNs, in which microtubule signals are spaced apart (fig. S18A). Furthermore, duolink proximity ligation assays (PLAs) supported the interaction between PR_20_ and KIF5B in both the cell body and processes of sMNs (fig. S18B). We also investigated the association of the transport machinery with endogenously expressed arginine-DPRs in postmortem tissue from patients with *C9-*ALS/FTD. We detected enrichment of KIF5B in 25 to 40% of cytoplasmic poly-GR inclusions in frontal cortex sections of all five patients analyzed (Fig. 4, F and G, and fig. S19A). Consistent with previous observations (*41*), GR inclusions were rare in spinal cord. The paucity of these structures in spinal cord prevented a meaningful quantification of their colocalization with KIF5B, although we did find instances of such events (fig. S19B). Collectively, our mass spectrometry, immunoprecipitation, and immunofluorescence data support physical association of arginine-rich DPRs with the axonal transport machinery.

### Microtubule-based transport is perturbed by arginine-rich DPRs in a reconstituted in vitro system

We next asked if arginine-rich DPRs directly impair the translocation of motors along microtubules using in vitro motility assays with purified components. Kinesin-1 and dynein were chosen for these experiments because, as introduced above, they are the major motors for mitochondrial transport in axons (*37*, *39*). These motors are also important for the transport of RNA granules in neuronal processes (*42*). To evaluate kinesin-1 motility, we used a well-characterized, constitutively active form of the major human isoform KIF5B tagged with GFP (*43*). Dynein motility assays were performed with a tetramethylrhodamine (TMR)–labeled human recombinant motor complex in the presence of the essential activators dynactin and BICD2N (*44*). Stabilized microtubules were bound to a glass surface and incubated with concentrations of motor complexes low enough for visualization of single transport events by total internal reflection fluorescence (TIRF) microscopy.

**Fig. 5:**
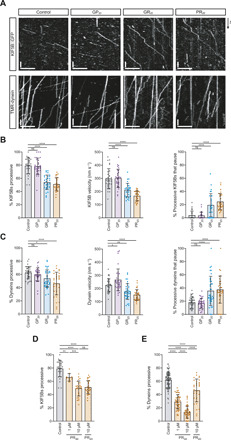
Arginine-rich DPRs perturb KIF5B and dynein motility in a reconstituted in vitro system. (**A**) Example kymographs of KIF5B and dynein in the presence of 10 μM GP_20_, GR_20_, PR_20_, or no peptide (control). The activating cofactors dynactin and BICD2N were present for all dynein experiments. Scale bars, 10 s and 5 μm; microtubule plus end to the right. (**B** and **C**) Effects of 10 μM GP_20_, GR_20_, or PR_20_ on percentage of microtubule-bound motors that are processive, velocity of processive movements, and percentage of processive complexes that undergo pausing. (**D** and **E**) Effects of poly-PR chain length and concentration on percentage of microtubule-bound motors that are processive. In (D) and (E), values for 10 μM PR_20_ and control are reproduced from (B) and (C) to facilitate comparison. In (B) to (E), means ± SD are shown; dots represent values for individual microtubules (see table S6 for numbers of microtubules and motor complexes analyzed). Statistical significance was evaluated with a one-way ANOVA multiple comparisons test with Dunnett’s correction (B and C) or Tukey’s correction (D and E) (*P < 0.05; **P < 0.01; ***P < 0.001; ****P < 0.0001). See figs. S20 and S21 for quantification of additional motile properties.

We first assessed the effects of 20-repeat DPRs on motility of KIF5B by introducing them into the motility assay together with the motor complex (Fig. 5A). GR_20_ and PR_20_ significantly reduced the percentage of microtubule-associated KIF5B complexes exhibiting processive transport compared to the control in which no peptides were added (Fig. 5B). These DPRs also caused a decrease in the mean velocity of processive KIF5B movements, as well as an increase in the incidence of pausing events within or at the end of a run (Fig. 5B). GR_20_ and PR_20_ had the same consequences on the motility of dynein in the presence of dynactin and BICD2N, although the magnitude of these effects relative to the no-peptide control tended to be smaller than observed for KIF5B (Fig. 5C). In contrast, GP_20_ did not reduce the percentage of processive events or velocity of either motor, or increase their incidence of pausing (Fig. 5, A to C).

Given the strong inhibition of mitochondrial transport in motor neurons by 30 repeats of PR (Fig. 2G and figs. S11 and S12), we also tested the effect of this peptide on purified KIF5B and dynein complexes. Compared to controls lacking peptide, PR_30_ impaired processive movements of both KIF5B and dynein complexes in a dose-dependent manner (Fig. 5, D and E, and fig. S20, A and B). The effect of PR_30_ on dynein was proportionally stronger than observed for PR_20_ (Fig. 5E and fig. S20C). In contrast, PR_30_ and PR_20_ impaired KIF5B motility to a similar extent (Fig. 5D and fig. S20D). Upon further analysis, we found additional differences in the effect of arginine-rich DPRs on KIF5B and dynein. First, there were more overall binding events of KIF5B on microtubules in the presence of GR_20_, PR_20_, and PR_30_ compared to no peptide controls (figs. S20D and S21A), whereas there were fewer such events for dynein relative to the control (figs. S20C and S21B). Second, while the arginine-rich DPRs prolonged the duration of processive movements for KIF5B, the opposite was true for processive dynein complexes (fig. S21). Collectively, our experiments demonstrate that arginine-rich DPRs perturb motility of both dynein and KIF5B in the absence of other potentially confounding cellular processes, although the effects of the peptides on the interactions of the two motors with microtubules are not equivalent.

### Microtubule-associated arginine-rich DPRs directly impede motor movement

**Fig. 6:**
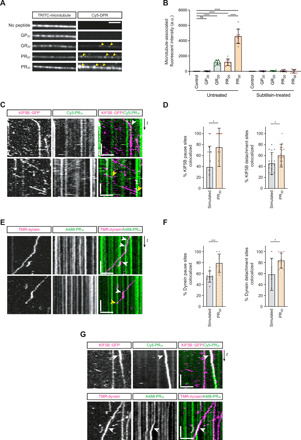
Arginine-rich DPRs bind microtubules and promote motor pausing and detachment. (**A**) Example images of microtubules incubated with arginine-rich DPRs. Arrowheads, intense peptide puncta. Scale bar, 2.5 μm. (**B**) Background-subtracted DPR signals ± subtilisin treatment. a.u., arbitrary units. (**C** to **F**) Analysis of localization of intense PR_30_ puncta with pausing or microtubule detachment events of motile motors (dynein experiments included dynactin and BICD2N). White and yellow arrowheads in kymographs show examples of, respectively, pausing and detachment events of motors localizing with PR_30_ foci. In (D) and (F), simulated controls were generated by overlaying motor trajectories with PR_30_ binding patterns from different microtubules or microtubule regions. (**G**) Kymographs showing examples (arrowheads) of translocation of PR_30_ puncta by motors. In all kymographs: scale bars, 10 s and 2.5 μm and microtubule plus end to the right. Chamber concentration of DPRs, 10 μM (A and B) and 1 μM (C to G). In (B), (D), and (F), means ± SD are shown; dots represent values for individual kymographs (see table S6 for number of pausing and detachment events analyzed). Statistical significance was evaluated with a one-way ANOVA multiple comparisons test with Tukey’s correction (B) or a Mann-Whitney *U* test (D and F) (*P < 0.05; ***P < 0.001; ****P < 0.0001).

To shed light on how arginine-rich DPRs impair motor movement, we monitored their localization in the in vitro motility assay. Consistent with our previous analysis using mouse spinal cord and human sMNs, there was a clear interaction of fluorescently labeled GR_20_, PR_20_, and PR_30_ with microtubules (Fig. 6A). All three peptides bound along the length of the lattice but were also concentrated in puncta (Fig. 6A). The strongest overall binding was exhibited by PR_30_ (Fig. 6B), which also had more frequent and more intense puncta than the 20-repeat DPRs (Fig. 6A). No microtubule association was observed for GP_20_ (Fig. 6, A and B). Thus, only those DPRs that perturb motility of KIF5B and dynein in the in vitro motility assay can associate with microtubules.

We next investigated which features of microtubules are required for binding to arginine-rich DPRs. Tubulin proteins comprise a folded core and a negatively charged, unstructured C-terminal tail that faces the bulk cytoplasm in the assembled microtubule. Microtubules formed from tubulin dimers with tails subsequently removed by the enzyme subtilisin (*45*) did not interact with poly-GR or poly-PR (Fig. 6B and fig. S22, A and B). This result demonstrates that the interaction of arginine-rich DPRs with microtubules is mediated by the tubulin tail.

These observations raised the possibility that tubulin tail–associated DPRs inhibit transport by acting as obstacles for KIF5B and dynein. Reducing the concentration of PR_30_ led to clearly resolvable puncta on microtubules (fig. S22C) that allowed us to investigate discrete interactions of DPRs and motor complexes on microtubules. We quantified the coincidence of sites of motor pausing or microtubule detachment with the patches of intense PR_30_ accumulation. To account for the probability that pausing events occurred at sites of PR_30_ foci by random chance, we overlaid motor trajectories on patterns of PR_30_ foci from different microtubules or microtubule regions from the same experiment (Fig. 6, C to F). These analyses showed that pause sites and detachment sites for both motors coincided with a patch of PR_30_ significantly more often than expected by random chance (Fig. 6, D and F). We conclude that large accumulations of arginine-rich DPRs can act as obstacles to KIF5B and dynein complexes. Our observation that the velocity of both motors along the microtubule was persistently reduced by poly-GR and poly-PR (Fig. 5, B and C) suggests that the more uniform binding of these DPRs along the rest of the lattice also impairs motility.

We additionally detected instances of each type of motor complex transporting large foci of PR_30_ along microtubules [Fig. 6G; observed for 1.8% of processive KIF5B complexes (3 of 169) and 8.1% of processive dynein complexes (9 of 111)]. These observations indicate that, in addition to associating with microtubules, PR_30_ can interact with KIF5B and dynein-dynactin-BICD2N complexes in our reconstituted system. This finding is consistent with our observation that arginine-rich DPRs can interact with both tubulin and motors in the cellular environment (Fig. 4, A to E).

**Fig. 7:**
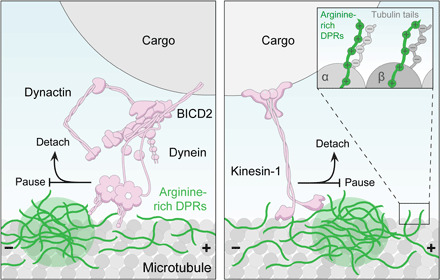
Working model for how arginine-rich DPRs inhibit microtubule-based motor complexes. The arginine-rich DPRs bind microtubules via the C-terminal tails of tubulin (right; inset). In addition to binding along the length of the microtubule lattice, the peptides concentrate in large, microtubule-associated foci (green circle). When dynein (shown here with dynactin and BICD2 as an example cargo adaptor) and KIF5B encounter these foci, interactions between the motors and arginine-rich DPRs facilitate motor arrest or detachment (in the crowded cellular environment, detached motors are also likely to be static). Molecular details of interactions between motor complexes and arginine-rich DPRs remain to be determined.

In summary, our single-molecule experiments reveal that interactions of arginine-rich DPRs with tubulin tails and motors impede transport along the microtubule track (schematized in Fig. 7). This mechanism offers an explanation for the decreased frequency of transport and increased incidence of pausing exhibited by motile mitochondria in neurons exposed to arginine-rich DPRs (Figs. 2 and 3) or derived from *C9*-ALS/FTD patients (Fig. 1).

## DISCUSSION

Axonal transport of organelles and macromolecules by microtubule motors is crucial for neuronal homeostasis and survival. This process facilitates communication between synaptic terminals and the cell body and allows local demands for the functions of organelles and macromolecules to be met (*35*). Inefficient microtubule-based transport in axons is linked with several age-related neurodegenerative diseases including Alzheimer’s, Huntington’s disease, and hereditary spastic paraplegia (*35*), as well as with ALS caused by mutations in *SOD1*, *TARDBP*, and *FUS* (*35*, *46*, *47*). However, whether impaired axonal transport is a cause or consequence of neuronal dysfunction in these contexts remains controversial.

The notion that defective intracellular trafficking can directly trigger ALS has been strengthened by the discovery of disease-causing mutations in several genes encoding components of the microtubule-based transport machinery, namely, *TUBA4A*, *DCTN1*, and *KIF5A* (*30*–*32*). These mutations have been reported to disturb the dynamics of microtubules or the trafficking of cargos along them (*48*). Despite evidence of defective axonal transport in other forms of ALS, the literature on *C9*-ALS/FTD has focused on other potential disease mechanisms (*5*). Here, we report that the disease-causing HREs in the *C9orf72* gene impair transport of mitochondria and RNA granules in neurites of human iPSC-derived sMNs. While our study was in review (and posted as a preprint), another group reported impaired microtubule-based trafficking of lysosomes in *C9orf72* neurons (*49*). Collectively, these data indicate that deficits in intracellular trafficking of cargos are a common feature of ALS pathogenesis.

Although both loss- and gain-of-function disease mechanisms have been proposed in *C9*-ALS/FTD (*5*, *49*), the transport impairment in our patient-derived motor neurons was not associated with a reduction in overall *C9orf72* mRNA and protein levels. However, we found that administration of the arginine-rich DPRs that are produced from *C9orf72* HREs dose dependently inhibits transport of mitochondria and RNA granules in sMNs derived from healthy individuals. Transport deficits become stronger with increasing numbers of repeats, raising the possibility that the longer DPRs that have been proposed to exist in patients (*50*) are more potent inhibitors of transport. We also observed that both poly-GR and poly-PR, but not poly-GA, reduced mitochondrial transport in axons of the intact adult *Drosophila* wing nerve. These observations, which are in line with the ability of PR_36_ to inhibit mitochondrial motility in the dissected *Drosophila* larval system (*51*), suggest a primarily gain-of-function mechanism in neurons involving inhibition of fundamental transport processes. The relevance of perturbed microtubule-based transport to the toxicity of arginine-rich DPRs is supported by our finding that reducing kinesin-1 gene dosage enhances the detrimental effects of arginine-rich DPRs in flies.

Our in vitro motility assays reveal that poly-GR and poly-PR can directly perturb microtubule-based transport of kinesin-1 and dynein, which are key motors in the trafficking of many cargos in neurons, including mitochondria and RNA granules. These peptides have a range of effects on motor behavior, including reducing the fraction of microtubule-associated complexes that are processive, as well as slowing the movement of complexes and increasing the likelihood of arresting during movement. We show that arginine-rich DPRs can associate with microtubules through an interaction with the C-terminal tubulin tails. The association of arginine-rich DPRs with the unstructured tubulin tails is in keeping with their propensity to bind intrinsically disordered regions in other proteins (*26*–*28*). Within the microtubule, tubulin tails face the bulk cytoplasm and participate in the interaction with dynein and kinesin motors (*52*). We show using purified proteins that arginine-rich DPRs bound to these sites act as obstacles for kinesin-1 and dynein, stimulating motor pausing or detachment (Fig. 7). Consistent with a physiological role for this mechanism, we found that arginine-rich DPRs also increase pausing of motile mitochondria in human sMNs and fly wing neurons. A similar increase in the incidence of mitochondrial pausing was also observed in sMNs derived from patients with *C9*-ALS/FTD, strengthening the evidence that inhibitory interactions of arginine-rich DPRs with the transport machinery are relevant for the disease.

The behaviors of arginine-rich DPRs on microtubules are reminiscent of the ability of the MAPT (tau) protein to partition into patches on microtubules and obstruct the motility of kinesin-1 and dynein complexes in vitro (*53*). Mutations in both *MAPT* and *C9orf72* have been shown to cause FTD, and it was recently proposed that this reflects a shared inhibitory effect on nucleocytoplasmic transport (*54*). The ability of both arginine-rich DPRs and MAPT to impede the motors responsible for trafficking in the cytoplasm offers another possible explanation for the overlapping features of *C9*-ALS/FTD and MAPT-FTD. Whether arginine-rich DPRs, like MAPT (*55*), can potentially also interfere with transport by modulating microtubule stability and dynamics is an interesting topic for future research.

We also detect physical association of arginine-rich DPRs with kinesin-1 and components of the dynein-dynactin transport complex, which could also contribute to the inhibition of transport. The reduced velocity and increased pausing of both types of motor in the presence of arginine-rich DPRs in vitro is compatible with their retardation by transient interactions with tubulin tail–associated peptides. Not all properties of KIF5B and dynein are affected equivalently by arginine-rich DPRs. While the peptides increase the frequency of KIF5B binding to microtubules and the duration of its processive runs, the opposite is true for dynein. These observations indicate that overall interactions of KIF5B and dynein complexes with microtubules are increased and decreased, respectively, by arginine-rich DPRs. While the mechanistic basis of this differential effect warrants further investigation, our results highlight how either strengthening or weakening interactions with microtubules can prevent efficient translocation of motors along the track.

Given the widespread roles of kinesin-1 and dynein in intracellular transport, as well as our discovery of other classes of microtubule motors in the PR_30_ interactome from sMNs, it is likely that the trafficking of several cargos is disrupted by arginine-rich DPRs. In line with this, we found that transport of both mitochondria and RNA granules is inhibited in the *C9orf72* lines and these changes are recapitulated in healthy sMNs treated with poly-PR and poly-GR. As described above, it was recently reported that lysosome trafficking is also impaired in neurons with the *C9orf72* HRE (*49*). Whether altered translocation of a specific cargo type is of particular significance for *C9-*ALS/FTD remains to be resolved. It is conceivable that additive effects from disrupting transport of multiple cargoes are at work.

While our results support a direct inhibitory effect of arginine-rich DPRs on axonal transport, it is possible that other processes also contribute to the trafficking defects we observe in *C9*-ALS/FTD neurons. In addition to defective nucleocytoplasmic transport, altered RNA splicing, gene expression, translation, and membrane-less organelle dynamics, as well as ER stress and bioenergetic defects of mitochondria, have been linked to the disease (*7*, *10*, *11*, *14*, *15*, *17*–*24*, *27*, *28*, *56*). The relative contribution of transport deficits versus other pathological effects of *C9orf72* mutations remains an open question. Addressing this issue will require the development of new tools to stimulate cargo transport in patient-derived neurons or abrogate arginine-rich DPR binding to tubulin tails. Nonetheless, our data raise the possibility that manipulating axonal transport machinery could be an effective strategy to hinder the progression of *C9*-ALS/FTD.

## MATERIALS AND METHODS

All experiments involving genetically modified organisms, animals, or human tissues were approved and overseen by the relevant Institutional Review Boards and complied with national regulations.

### Cell culture and motor neuron differentiation

*C9orf72* iPSC lines were generated from dermal fibroblasts from ALS (C9-2B and C9-3B) and ALS/FTD (C9-1B and C9-4B) patients carrying the G_4_C_2_ repeat expansion in the *C9orf72* gene (for clinical information, see table S1). Skin fibroblasts were obtained from patients and controls after informed consent and approval by the Ethics Committee of the University Hospitals Leuven. Controls were generated from unrelated healthy individuals. Con 1B and Con 2B were purchased from Takara Bio Inc. (ChiPSC-6b, P11031) and Sigma-Aldrich (iPSC Epithelial-1, IPSC0028), respectively. Con 3B, C9-1B, C9-2B, C9-3B, and C9-4B were generated using the CytoTune-iPS 2.0 Sendai Reprogramming Kit (Thermo Fisher Scientific). qRT-PCR for Sendai virus clearance was performed, and no residual Sendai virus RNA was detected in any of the tested iPSC lines. iPSCs were kept in Essential 8 flex medium (Gibco) with penicillin-streptomycin (1000 U/ml). Colonies were passaged every week with 0.5 mM EDTA (Invitrogen) diluted in Dulbecco’s phosphate-buffered saline (DPBS; Sigma-Aldrich) and plated on Geltrex LDEV-Free, hESC-Qualified, Reduced Growth Factor Basement Membrane Matrix (Gibco). The absence of mycoplasma contamination was routinely confirmed by PCR. Controls (Con-1B, Con-2B, and Con-3B) and *C9orf72* (C9-1B, C9-2B, C9-3B, and C9-4B) iPSCs were differentiated into sMNs as previously described (*46*, *57*). The controls (Con-1 and Con-2), the isogenic controls (C9-1∆, C9-2∆, C9-3∆), and the *C9orf72* counterparts (C9-1, C9-2, and C9-3) were obtained and differentiated as reported by Selvaraj *et al.* (*33*).

### Immunocytochemistry

Coverslips were incubated for 20 min at room temperature in PBS with 4% paraformaldehyde and washed three times with PBS. Cells were incubated for 1 hour at room temperature in PBS containing 0.1% Triton X-100 (Acros Organics) and 5% donkey (Sigma-Aldrich) or goat (Dako) serum. Cells were incubated with primary antibodies diluted in PBS containing 0.1% Triton X-100 and 2% serum overnight at 4°C. Cells were rinsed three times in PBS before the secondary antibody was added for 1 hour at room temperature. See table S7 for details of primary and secondary antibodies. After washing with PBS, the coverslips were incubated for 20 min in PBS/4′,6-diamidino-2-phenylindole (DAPI) (NucBlue Live ReadyProbes Reagent, Invitrogen). Coverslips were rinsed three times with PBS before mounting in ProLong Gold antifade reagent (Invitrogen). Confocal images were obtained with a Leica SP8 DMI8 confocal microscope. Images were analyzed, formatted, and quantified with ImageJ software.

### Pluripotency characterization

iPSCs were subjected to spontaneous differentiation mediated by the formation of embryoid bodies (EBs) and subsequently analyzed for three-lineage differentiation using the ScoreCard methodology (Thermo Fisher Scientific). On the day of EB formation, 60 to 80% confluent iPSCs were washed with PBS and incubated with 0.5 mM EDTA (Invitrogen) for 1 to 3 min to dissociate the colonies. Cells were harvested in Essential 8 flex medium (Gibco) and counted before gently spinning down at 300*g* for 5 min. Cells were plated in a 24-well Corning Ultra-Low Attachment Surface plates (Corning) at a density of 1.5 × 10^6^ cells per well. The plates were incubated overnight at 37°C. The following day, half of the Essential 8 flex medium was replaced with Essential 6 medium (Gibco). For the spontaneous differentiation, EBs were kept for 14 days in E6 medium, which was changed every 2 days. After 14 days in culture, EBs were collected for RNA extraction with the GenElute Mammalian Total RNA Kit (Sigma-Aldrich). Complementary DNA (cDNA) synthesis was performed with SuperScript III (Thermo Fisher Scientific), and qPCR was performed according to the manufacturer’s protocol with TaqMan hPSC Scorecard assay (Thermo Fisher Scientific). Data were analyzed with the online Scorecard software (Thermo Fisher Scientific).

### Real-time PCR

Total RNA extraction was performed with the GenElute Mammalian Total RNA Kit (Sigma-Aldrich). cDNA was synthesized from 1 μg of total RNA using SuperScript III First-Strand Synthesis SuperMix for qRT-PCR (Invitrogen) according to the manufacturer’s instructions. The qPCR for Sendai virus detection was performed using TaqMan Gene Expression Assays (Life Technologies). Quantitative RT-PCR was performed using SYBR Green PCR Master Mix (Applied Biosystems) on the 7500 Step OnePlus Real-Time PCR System (Applied Biosystems). Relative gene expression was determined by the 2^−ΔΔ*C*t^ method with normalization to *GAPDH* mRNA.

### Repeat-primed PCR

The *C9orf72* repeat expansion mutation was confirmed in both *C9-*ALS/FTD iPSCs and MNs by repeat-primed PCR (RP-PCR). First, the G_4_C_2_ hexanucleotide repeat was amplified using the forward primer *C9orf72*-PCR-F (6-FAM) CAAGGAGGGAAACAACCGCAGCC and the reverse primer *C9orf72*-PCR-R GCAGGCACCGCAACCGCAG. The PCR mix was prepared in a 25-μl solution consisting of 100 ng of genomic DNA, 10× PCR amplification buffer, 0.5 μl of Taq DNA polymerase (Roche), 2.5 μM forward primer, 2.5 μM reverse primer, 2 mM deoxyribonucleotide triphosphate (dNTP) (Amersham Biosciences), 50 mM MgSO_4_ (Invitrogen), 6 μl of PCR enhancer solution (Invitrogen), and dH_2_O to the final volume. After a 5-min incubation at 94°C, the reactions were subjected to 28 cycles of 95°C for 30 s, 55°C for 30 s, 68°C for 60 s, followed by a 5-min extension at 68°C. The forward primer *C9orf72*_F_FAM_bis (6-FAM)-AGTCGCTAGAGGCGAAAGC, the reverse primer *C9orf72*_R1_bis TACGCATCCCAGTTTGAGACGGGGGCCGGGGCCGGGGCCGGGG, and the second reverse primer *C9orf72*_R2_bis TACGCATCCCAGTTTGAGACG were used for the amplification of the G_4_C_2_ hexanucleotide repeat. The RP-PCR assay was performed in a reaction volume of 25 μl containing 100 ng of genomic DNA, 10× Expand long template buffer 2 (Roche), Expand long template enzyme mix (Roche), 2.5 μM primer F, 2.5 μM primer R1, 2.5 μM primer R2, 20 mM dNTPs (Amersham Biosciences), 5 mM betaine (Sigma-Aldrich), and dH_2_O to the final volume. After a 10-min incubation at 98°C, the reactions were subjected to 10 cycles of denaturation at 97°C for 35 s, 53°C for 2 min, 68°C for 2 min, followed by 25 cycles (+20 s per cycle) of 97°C for 35 s, 53°C for 2 min, and 68°C for 2 min. This step was followed by a 10-min extension at 68°C. PCR products were incubated at 95°C for 3 min and cooled on ice followed by 5 μl of each PCR product being added to 18 μl of formamide (Sigma-Aldrich) and 2 μl of Genescan 500 Rox Size Standard (Applied Biosystems). Fragment length analysis was performed with GeneMapper software version 4.0 (Applied Biosystems) following electrophoresis on an automatic sequencer (DNA Analyzer; Applied Biosystems). A characteristic stutter amplification pattern on the electropherogram was considered diagnostic of a pathogenic repeat expansion.

### Southern blotting

Southern blots were performed as described previously (*58*). Briefly, genomic DNA was extracted using the Wizard Genomic DNA Purification Kit (Promega) and digested with the Xba I restriction enzyme (Promega). After electrophoresis for 6 hours at 100 V on a 0.8% agarose gel, DNA was transferred to a positively charged nylon membrane (Roche) and then cross-linked by ultraviolet (UV) irradiation. Hybridization was performed with a digoxigenin (DIG)–labeled probe (Roche). The probe was detected with an anti-DIG antibody (Roche; 1:10,000) and visualized using CDP-Star substrate (Roche) and autoradiography film.

### Neurite outgrowth assay

Time-lapse imaging of neurite outgrowth was performed using the IncuCyte Zoom system (Sartorius). sMNs were plated on 24-well plates at day 10 of the motor neuron differentiation at a density of ~20,000 cells per well. A minimum of four bright-field images were taken per day using a 20× Plan Fluor objective [0.45 numerical aperture (NA), Nikon] from days 14 to 18. The lengths of neurites were automatically measured using the IncuCyte Zoom Neurotrack software module.

### Electrophysiology

Whole-cell patch-clamp recordings from day 38 iPSC-derived motor neurons were performed at room temperature in artificial cerebrospinal fluid (aCSF) consisting of 140 mM NaCl, 5 mM KCl, 2 mM CaCl_2_, 2 mM MgCl_2_, 10 mM Hepes, and 12 mM glucose (pH 7.4; ~300 mOsm). Borosilicate glass patch pipettes were pulled by a vertical PIP6 micropipette puller (HEKA Elektronik) and filled with an internal solution containing 120 mM K-gluconate, 20 mM KCl, 1 mM MgCl_2_, 10 mM Hepes, 0.2 mM EGTA, 0.3 mM Na-GTP (guanosine triphosphate), 5 mM NaCl, and 4 mM Mg-ATP (adenosine triphosphate) (pH 7.3; ~290 mOsm). Motor neurons were visualized with an inverted Olympus IX73 microscope equipped with a 40× objective (0.60 NA, Ph2). Action potentials were recorded in current-clamp mode. Signals were acquired, filtered (at 2.8 kHz), and digitized (at 20 kHz) using an EPC10 USB amplifier and PatchMaster software (HEKA Elektronik). The liquid junction potential of 13.8 mV was corrected offline. Action potentials were detected using Stimfit software.

### Digital droplet PCR

The QX200 Droplet Digital PCR (ddPCR) system (Bio-Rad) was used to provide absolute measurements of *C9orf72* mRNA, as well as each of the V1, V2, and V3 transcript variants. Total RNA was extracted with the RNeasy Mini Kit (Qiagen) following the manufacturer’s instructions. The cDNA was obtained using the SuperScript III First-Strand Synthesis System for RT-PCR kit (Thermo Fisher Scientific) according to the manufacturer’s instructions. Digital droplet PCR was performed following guidelines from Bio-Rad. Briefly, each ddPCR mix was prepared in a 22-μl solution consisting of 2× ddPCR Supermix for probes (without deoxyuridine triphosphates), 20× FAM TaqMan probe, 20× Hex TaqMan probe, 4 μl of diluted cDNA (1:10), and 4.8 μl of nuclease-free dH_2_O (Thermo Fisher Scientific). Droplets were generated in the QX200 Droplet Generator and transferred to a standard 96-well PCR plate that was heat-sealed using foil sheets (Pierceable Foil Heat Seal, Bio-Rad) and a PX1 PCR plate sealer. The droplets were amplified using a Bio-Rad T100 thermal cycler starting with 10-min enzyme activation at 95°C, followed by 40 cycles of 95°C, 30 s and 60°C, 1 min and a final hold at 98°C for 10 min (2°C/s ramp rate). The fluorescence of each thermal cycled droplet was measured using the QX200 Droplet Reader (Bio-Rad), and the results were analyzed with QuantaSoft software (Bio-Rad) after threshold setting on fluorescence of negative controls. The number of positive and negative droplets was used to calculate the concentration (cDNA copies/μl of the final 1× ddPCR) of the targets and their Poisson-based 95% confidence intervals. A reference gene, *SCLY*, was included to increase the accuracy of the relative gene expression. Graphs show relative expression levels. The V1 (6-FAM-TAA TGT GAC-ZEN-AGT TGG AAT GC-IBFQ), V2 (6-FAM-CGG AGC ATT-ZEN- GG ATA ATG TGA CAG TTG G-IBFQ), V3 (6-FAM-TAA TGT GAC-ZEN-AGT TGG AAT GC-IBFQ), and Vall (6-FAM-ACA GAG AGA-ZEN-ATG GAA GAT CAG GGTC AGA-IBFQ) probes were used in this experiment (6-FAM, 6-carboxyfluorescein; ZEN, internal quencher; IBFQ, Iowa Black dark fluorescence quencher).

### Analysis of C9orf72 protein levels

Cell pellets were lysed by resuspension in radioimmunoprecipitation assay (RIPA) buffer [50 mM tris-HCl (pH 7.5), 150 mM NaCl, 1% NP-40, 0.5% Na-deoxycholic acid, 0.5% SDS] supplemented with cOmplete EDTA-free Protease Inhibitor Cocktail (Sigma-Aldrich). The samples were centrifuged at 14,000 rpm for 20 min, and the supernatant was transferred to a prechilled tube. Total protein concentrations were quantified using the Micro BCA Protein Assay Kit (Thermo Fisher Scientific) according to the manufacturer’s instructions. Equal amounts of proteins were boiled at 95°C with 5× reducing sample buffer (Thermo Fisher Scientific) and separated by SDS–polyacrylamide gel electrophoresis (SDS-PAGE). After electrophoresis, the proteins were transferred to a polyvinylidene difluoride (PVDF) membrane (Trans-Blot Turbo Mini 0.2 μm PVDF, Bio-Rad) using the Trans-Blot Turbo Transfer System (Bio-Rad, Mixed MW program, 25 V, 1.3 A, 7 min). The membranes were blocked with 5% nonfat dry milk diluted in tris buffer saline solution with Tween [TBS-T; 1 M tris-HCl (pH 7.5), 150 mM NaCl, 0.1% Tween 20] for 1 hour at room temperature followed by overnight incubation at 4°C with anti-C9orf72 primary antibody (table S7) diluted in TBS-T. For the loading control signals, the membranes were subsequently incubated for 2 hours at room temperature with anti-Calnexin primary antibody (table S7) diluted in TBS-T. Following incubation with the primary antibodies, membranes were washed three times with TBS-T and incubated for 1 hour at room temperature with secondary antibodies coupled to horseradish peroxidase [anti-mouse or anti-rabbit, 1:5000, Agilent Technologies (Dako)]. After three washes with TBS-T and one wash with TBS, chemiluminescence signals were generated by ECL Western Blotting Substrate (Thermo Fisher Scientific) or SuperSignal West Femto Maximum Sensitivity Substrate (Thermo Fisher Scientific) and detected with the ImageQuant LAS 4000 Biomolecular Imager (GE Healthcare Life Sciences). Band intensities were quantified using ImageQuant TL version 7.0 software (GE Healthcare Life Sciences) and normalized to the Calnexin loading control.

### Enzyme-linked immunosorbent assay

Poly-GP and poly-GA levels in control and *C9orf72*-derived sMN lysates were measured using a sandwich immunoassay that uses Meso Scale Discovery (MSD) electrochemiluminescence detection technology. Lysates were diluted in assay diluent (1% Blocker-A/PBST), and equal amounts of protein for all samples were tested for poly-GP. The assay uses an affinity-purified goat polyclonal antibody to poly-GP (Biogen-3746, 1 μg ml^−1^) as capture, and mouse monoclonal poly-GP antibody (Biogen-2P8H9.1.1, 0.5 μg ml^−1^) along with a SULFO-tag anti-mouse secondary antibody (1 μg ml^−1^), to detect captured poly-GP. Similarly, lysates were diluted in assay diluent (1% Blocker-A/PBST) and equal amounts of protein for all samples were tested for poly-GA using an affinity-purified goat polyclonal antibody to poly-GA (Biogen-3682, 1 μg ml^−1^) as capture, and mouse monoclonal to poly-GA antibody (Biogen-2P36E2.1.1, 1 μg ml^−1^) along with a SULFO-tag anti-mouse secondary antibody (1 μg ml^−1^). Each sample was tested in duplicate wells, sample volume permitting, using 50 μl per well. Each assay plate contained the same control samples (serial dilutions of iPSC motor neuron cell lysates derived from a *C9orf72* mutation carrier and a noncarrier, and serial dilutions of synthetic GP_8_ or GA_8_). The reported response values correspond to intensity of emitted light upon electrochemical stimulation of the assay plate using MSD Quickplex SQ120 after subtraction of the background signal in wells containing only assay buffer.

### Cell viability assay

Cell viability after DPR treatment was measured using DRAQ7 (Abcam) far-red fluorescent DNA dye, a cell impermeable agent that only stains the nuclei in cells with a compromised cell membrane (i.e., that are in the process of dying). DRAQ7 was added to each well at a concentration of 3 μM together with Hoechst 33342 (1:1000, Thermo Fisher Scientific) and incubated for 20 min at 37°C. Cells were then washed once with Dulbecco’s modified Eagle’s medium (DMEM)/F12 and fixed with 4% paraformaldehyde in PBS for 20 min. For this experiment, motor neurons were cultured in a CellCarrier 96-well plate (PerkinElmer). Motor neurons were treated with the respective synthetic peptides (PR_20_, GR_20_, and GP_20_; Pepscan), and the analysis was performed 16 hours after treatment. Untreated cells were used as the control group for PR_20_ and GR_20_. To allow the uncharged GP_20_ peptide to pass the cellular membrane, cells were treated with streptolysin O (SLO; 15 ng ml^−1^, Sigma-Aldrich) diluted in Hanks’ balanced salt solution (HBSS) (without Ca^2+^, Gibco) containing 30 mM Hepes (Sigma-Aldrich) for 15 min at 37°C. In this case, cells treated with SLO without GP_20_ peptide were used as untreated controls. Images were acquired using the Operetta CLS High Content Screening (HCS) System (PerkinElmer, UK) using a 20× air objective lens (0.4 NA, correction collar). Seventy-seven fields of view were acquired per well. Images were analyzed using Harmony software (PhenoLOGIC, PerkinElmer).

### Visualization and analysis of cargo transport in human motor neurons

Motor neuron cultures were stained with MitoTracker Red (50 nM, Invitrogen) or SYTO RNASelect Green fluorescent (500 nM, Invitrogen) for 20 min at 37°C, according to the manufacturer’s instructions. Control sMNs were treated with the respective synthetic peptides for 16 or 72 hours before analysis of cargo transport, as described for the cell viability assay. Live cell imaging was performed using an inverted Zeiss Axiovert 200M microscope (Carl Zeiss) with a 40× water immersion lens (1.2 NA, C-Apochromat). During the recording, the cells were perfused with Hepes solution (10 mM Hepes, 148 mM NaCl, 5 mM KCl, 0.1 mM MgCl_2_, 10 mM glucose, and 2 mM CaCl_2_) and maintained at a temperature of 36 ± 0.5°C using a gravity-fed and heated perfusion system (custom built). A TILL Poly V light source (Till Photonics, Graefelfing, Germany) was used to excite MitoTracker (540 nm) and RNASelect (475 nm), and 200 images at 1 Hz were recorded by a cooled charge-coupled device (CCD) camera (PCO sensicam-QE) using TILL VisION (TILL Photonics) software. All image analysis was performed in Igor Pro (Wavemetrics, Portland, USA) using custom-written routines (*59*). Neurites with at least one moving organelle were considered for the analysis. Kymographs were generated for each neuronal process to distinguish stationary mitochondria (visible as vertical lines) and moving mitochondria (visible as diagonal lines). The numbers of stationary and moving mitochondria were calculated by counting straight and diagonal lines on the kymographs.

When assessing pausing of motile mitochondria in sMNs, movies were stabilized if required using the ImageJ plugin Stackreg. Kymographs were generated along neurites, and all mitochondrial movements were examined for pausing. Pauses within or at the end of runs were scored only if the stationary event exceeded 3 s (>3 frames). Blinded analysis was carried out to avoid introducing unconscious bias.

To determine the directionality of transport, cells were sparsely transduced with lentivirus expressing mitoDsRed2 at day 16 of the sMN differentiation using a multiplicity of infection (MOI) of 0.5, optimized to visualize one to two labeled cells per field of view. Imaging of mitochondrial axonal transport was performed at day 38 of sMN differentiation with a 63× oil immersion lens [Plan-Apochrat 1.40 NA (DIC M27) objective, Carl Zeiss] using an Axio Observer Z1 inverted motorized microscope (Carl Zeiss) equipped with an Cy3 FL filter set (Carl Zeiss), Zen 2011 z-stack, time-lapse and definite-focus modules (Carl Zeiss), and an S1 Environmental System incubation chamber (Carl Zeiss) maintained at 37°C and 5% CO_2_. Medium was changed 30 min before imaging to phenol red–free Neurobasal medium supplemented with 1× GlutaMAX. Mitochondrial movements were recorded for 5 min using a 0.2-Hz capture of a 100-μm stretch of neurites and a small z-stack. At least four neurites were imaged per cell line per differentiation. sMN genotypes were counterbalanced and interleaved between experimental runs. Maximum intensity projections were computed in Fiji. Kymographs were generated and analyzed using KymoToolBox in Fiji to determine the numbers of stationary (≤0.1 μm/s) versus motile mitochondria and the predominant directionality of movement [either away from (anterograde) or toward (retrograde) the soma].

### *Drosophila* husbandry and strains

Flies were fed with Iberian food [5.5% (w/v) glucose, 5% (w/v) baker’s yeast, 3.5% (w/v) organic flour, 0.75% (w/v) agar, 16.4 mM methyl-4-hydroxybenzoate, 0.004% (v/v) propionic acid] and maintained at 25°C on a 12-hour-light, 12-hour-dark cycle at 50 ± 5% humidity. The following strains were used: *yw* [control (Bullock laboratory stocks)]; *w^1118^* [control (Bullock laboratory stocks)]; *GMR-Gal4 UAS-GR_36_/CyO* and *GMR-Gal4 UAS-PR_36_/CyO* (*9*); *FRT G13 Khc^27^/CyO* and *b^1^ pr^1^ Khc^8^/CyO DGY* (*Khc^null^ alleles*) (*60*); *Dhc^STOP^/TM6B* [*Dhc^null^* allele (contains a CRISPR-induced out-of-frame indel that causes a premature stop at codon 169) (wild-type protein has 4638 codons) (see below)]; *dpr-Gal4; UAS-mito::GFP/CyO-TM6B* (*61*); and *UAS-GA_36_*, *UAS-GA_100_*, *UAS-GR_36_*, *UAS-GR_100_*, *UAS-PR_36_*, and *UAS-PR_100_* (*9*). For the experiment in fig. S16C, the *Khc^27^* allele was outcrossed into a *w^1118^* background for seven generations before rebalancing using a *yw; Pin/CyO* stock. The control stock for this experiment was made using the *Khc^+^/CyO* siblings from the rebalancing cross. The *Dhc^null^* allele was generated as described (*62*) by injecting embryos of the *nos-cas9* strain with a pCFD3 plasmid expressing a guide RNA (gRNA) with the following target site: CGCGGCCTGGTCGTGGAGG. The mutation was then outcrossed to a *w^1118^* background for seven generations before balancing with a *yw;; MKRS/TM6B* stock.

### Assessment of mitochondrial trafficking in the *Drosophila* marginal wing nerve

*dpr-Gal4; UAS-mito::GFP/CyO-TM6B* males were crossed to virgin females of the following genotypes: *yw (control)*, *UAS-GA_36_*, *UAS-GA_100_*, *UAS-GR_36_*, *UAS-GR_100_*, *UAS-PR_36_*, and *UAS-PR_100_.* Recently eclosed male *dpr-Gal4*, *UAS-mito::GFP* offspring with and without *UAS-‘DPR’* transgenes were collected and aged for ~48 hours. Male flies were used for all phenotypic analysis to control for any sex-specific phenotypic effects. Flies were anesthetized with CO_2_ (0.8 bar) for 5 min and then mounted on No.1 coverslips with their wings gently spread out in a thin layer of 10S Voltaleff halocarbon oil (VWR), as described (*36*). Movies were typically acquired from both wings at 0.5 Hz for 180 s using a single-channel acquisition at 488 nm and processed and analyzed in Fiji using the CellCounter and MTrackJ plugins. Movies were acquired on a PerkinElmer Spinning Disk confocal using a 60× oil-immersion objective (PlanApo, 1.4 NA) and an Olympus IX71 microscope. Movies were oriented so that cell bodies (retrograde direction) were on the left and axon termini (anterograde direction) were on the right. Movies were stabilized using StackReg (Rigid body) and straightened using the in-built Fiji Straighten tool before being cropped to display 50 μm of the wing nerve. Processed movies were analyzed blind to the genotype. Mitochondria number was defined as average number of mitochondria when counted at the 1st, 46th, and 91st frames (counted with the CellCounter plugin). Mitochondrial movements were tracked using the MTrackJ plugin in Fiji. Movements were considered a transport event if the total displacement exceeded 2 μm. A transient stationary period of a motile mitochondrion was scored as a “pause” if it lasted ≥2 frames (≥4 s). Results were exported from CellCounter and MTrackJ into Excel and plotted in Prism 8 (GraphPad).

### Assessment of *Drosophila* viability and eye defects

Male *GMR-Gal4 UAS-GR_36_* or *GMR-Gal4* *UAS*-*PR_36_* flies were mated with females with *Khc* or *Dhc* null alleles or *yw* controls to study the effects of motor gene dosage on DPR toxicity. Although *GMR-Gal4* is predominantly expressed in the eye, its ability to cause lethality in combination with *UAS-GR_36_* (*9*) indicates that it has some activity in other tissues (the eye is not required for survival to adulthood). Each type of cross was set up in triplicate, with at least two egg lays (of 3 days each) analyzed per cross. Adult offspring were collected and aged to 4 to 6 days after eclosion before analysis. For all crosses that produced a sufficient number of offspring (>15 internal control animals that did not inherit the *GMR-Gal4 UAS-GR_36_* chromosome), the number of male flies of different genotypes that inherited the *GMR-Gal4 UAS-GR_36_* chromosome was counted. These values were plotted as a percentage of the expected number of offspring for each genotype assuming no lethality, based on the number of control offspring that did not inherit the *UAS-GR_36_* chromosome. To account for the contribution of genetic background, both an outcrossed *Khc^27^* line and an independent null allele of *Khc* were also assessed for lethality (see the “*Drosophila* husbandry and strains” section).

For analysis of eye defects, a single eye per *GMR-Gal4 UAS-PR_36_* male was scored using a qualitative scale of eye disruption (no, mild, moderate, and severe; table S4). Necrotic patches were not classed as pigmentation. See fig. S16A for example images for each category. All eye scoring was carried out blind of the genotypes. In control experiments, we confirmed that decreased motor gene dosage does not affect survival or eye morphology in the absence of *GR_36_* or *PR_36_* expression (table S4).

### *Drosophila* eye imaging

Images exemplifying the different classes of eye phenotype were acquired using a Nikon “Multiphot” macrophotography setup, with a 6× Macro-Nikkor 35-mm lens and a Sony a6300 “mirrorless” 24-megapixel camera, followed by processing in Adobe Photoshop CS6.

### Mouse procedures and husbandry

For spinal cord dissection, C57BL/6 mice (The Jackson Laboratory) were anesthetized with isoflurane and transcardially perfused with 0.9% saline. Isolated spinal cords were used immediately for sample preparation. All mouse procedures and husbandry were performed in accordance with Institutional Animal Care and Use Committee guidelines and approved by the Stanford Administrative Panel on Animal Care.

### PR_30_ protein precipitation

Mouse spinal cords were disintegrated in PBS buffer with Halt Protease inhibitor (Thermo Fisher Scientific) with a Dounce homogenizer. The cell suspension was subsequently lysed on ice using a probe sonicator (Branson Sonifier 250, VWR Scientific). The soluble lysate was cleared from the insoluble fraction by centrifugation for 15 min at 10,000 rpm (11,292*g*) at 4°C (Eppendorf 5427 R). The supernatant was retrieved, and the procedure was repeated until no pellet was visible after centrifugation. The protein concentration was measured using the Micro BCA assay (Thermo Fisher Scientific). PR_30_ (Pepscan) was added to a final concentration of 50 μM to 0.5 mg of soluble lysate in a total volume of 400 μl, and samples were incubated for 15 min. The volume of the samples was increased to 1.5 ml with PBS before gently spinning down the PR droplets at 4000 rpm (3220*g*) for 5 min (Eppendorf 5810 R). Eppendorf tubes with phase-separated samples were dispensed in 50 ml Falcon tubes and spun down in a swinging-bucket centrifuge. Pellets were subsequently washed with 1 ml of PBS and vortexed before spinning down again. Washing steps were repeated three times. The resulting pellets were processed for liquid chromatography–tandem mass spectrometry (LC-MS/MS).

For cross-linked samples (see table S5), after incubation with PR_30_, paraformaldehyde (Sigma-Aldrich) was added to a final concentration of 0.5% for 5 min. Five hundred microliters of 2 M glycine (Sigma-Aldrich) was then added for 5 min to quench paraformaldehyde. Samples were subsequently treated the same as uncross-linked samples. Samples for analysis by SDS-PAGE and silver staining were processed identically, with silver staining performed according to the manufacturer’s instructions (Thermo Fisher Scientific).

### Proteomics sample preparation and LC-MS/MS analysis

Pellets were redissolved in 8 M urea in PBS and solubilized by sonication, with a total sample volume of 1.5 ml. Proteins in each sample were reduced with 5 mM dithiothreitol (DTT) (30-min incubation at 55°C) and alkylated by addition of 10 mM iodoacetamide for 15 min at room temperature in the dark. Samples were further diluted to give a final urea concentration of 2 M, and proteins were digested with trypsin (1:100, w/w) (Promega) overnight at 37°C. Peptides were purified on Omix C18 tips (Agilent) and dried and redissolved in 25 ml of solvent A [0.1% trifluoroacetic acid (TFA) in dH_2_O/acetonitrile (98:2, v/v)]. Ten milliliters of this material was injected for LC-MS/MS analysis on the Ultimate 3000 RSLCnano System (Dionex, Thermo Fisher Scientific) in-line connected to a Q Exactive HF mass spectrometer fitted with a Nanospray Flex Ion source (Thermo Fisher Scientific). Trapping was performed at 10 ml/min for 4 min in solvent A (on a reversed-phase column produced in-house, 100-mm inside diameter (ID) × 20 mm, 5-mm beads C18 Reprosil-Pur, Dr. Maisch). The sample was then loaded on a 40-cm column (produced in-house, 75-mm ID × 400 mm, 1.9-mm beads C18 Reprosil-HD, Dr. Maisch). Peptides were eluted using an increase in solvent B [0.1% formic acid in dH_2_O/acetonitrile (2:8, v/v)] in linear gradients from 2 to 3% over 100 min, then from 30 to 56% over 40 min, and from 56 to 99% from 5 min (all with a constant flow rate of 250 nl/min). The mass spectrometer was operated in data-dependent mode with automatic switching between MS and MS/MS acquisition for the 16 ion peaks per spectrum with the highest abundance. Full-scan MS spectra [375 to 1500 mass/charge ratio (*m/z*)] were acquired at a resolution of 60,000 after accumulation to a target value of 3,000,000 (maximum fill time, 60 ms). The 16 most intense ions above a threshold value of 22,000 were isolated (window of 1.5 Th) for fragmentation at a normalized collision energy of 32% after filling the trap at a target value of 100,000 for a maximum of 45 ms. The S-lens RF level was set at 55, and precursor ions with single and unassigned charge states were excluded.

Data analysis was performed using MaxQuant software (version 1.5.3.30) with the Andromeda search engine with default search settings (including a 1% false discovery rate at both the peptide and protein level). Spectra were searched against the mouse proteins in the UniProt/Swiss-Prot database (database release version of April 2016 containing 20,103 mouse protein sequences, www.uniprot.org). For precursor and fragment ions, mass tolerances were set to 4.5 and 20 parts per million, respectively, during the main search. Enzyme specificity was set as C-terminal to arginine and lysine, with cleavage at proline bonds also permitted, with a maximum of three missed cleavages. Variable modifications were set to acetylation of protein N termini and oxidation of methionine residues. Only proteins with at least one unique or razor peptide were retained, which lead to identification of 1811 mouse proteins. Proteins were quantified using the MaxLFQ algorithm integrated in the MaxQuant software (*63*). Quantification required a minimum ratio count of two unique or razor peptides. Perseus software (version 1.5.3.0) was used for further data analysis after loading the MaxQuant protein groups file. Proteins identified only by site and reverse database hits were removed. Missing values were produced by imputation from a normal distribution around the detection limit.

### Coimmunoprecipitation

Motor neurons were treated with 10 μM of HA-labeled DPR peptides (PR_20_, GR_20_, and GP_20_; Pepscan) for 3 hours. Subsequently, medium was removed and washed once with ice-cold DPBS (Sigma-Aldrich). Motor neurons were gently scraped off the dish and centrifuged for 5 min, 400*g* at 4°C. Cell pellets were cross-linked using 1% paraformaldehyde (Sigma-Aldrich) in DPBS and incubated for 10 min at room temperature. The cross-linking reaction was stopped with ^1^/_10_ volume of 1.25 M glycine (pH 8) (Thermo Fisher Scientific). Cross-linked cell pellets were washed three times with ice-cold DPBS followed by lysis or snap freezing and storage at −80°C. Cell pellets were lysed in ice-cold lysis buffer [150 mM NaCl, 25 mM tris, 1 mM EDTA, 1% NP-40, and 5% glycerol (pH 7.4)] supplemented with complete EDTA-free protease inhibitor cocktail (Roche Diagnostics). Cell debris was removed via centrifugation, and supernatants were mixed with Pierce anti-HA magnetic beads (Thermo Fisher Scientific). The mixture was incubated for 1 hour at room temperature with agitation. Afterward, the beads were washed three times with lysis buffer. The HA-tagged dipeptides and associated material were eluted using the manufacturer’s buffers.

For immunoblot analysis, samples were resolved on 4 to 20% mini-protean TGX stain-free gels (Bio-Rad) and transferred to nitrocellulose membrane (Bio-Rad). Membrane was blocked with 5% nonfat milk (Bio-Rad) in TBS-T for 2 hours at room temperature and incubated with primary antibodies overnight at 4°C (table S7). The next day, the membranes were washed three times with TBS-T and incubated for 1 hour with secondary Trueblot antibodies (Rockland). Proteins were detected by enhanced chemiluminescence reagents (Thermo Fisher Scientific) and detected with the ImageQuant LAS 4000 Biomolecular Imager (GE Healthcare Life Sciences).

### Proximity ligation assay

Duolink PLA (Sigma-Aldrich) was performed according to the manufacturer’s instructions. Briefly, samples were fixed with 4% paraformaldehyde in PBS for 20 min, rinsed three times, and incubated for 1 hour at room temperature in PBS containing 0.2% Triton X-100 and 5% donkey serum. Cells were incubated overnight at 4°C in 2% donkey serum containing the primary antibodies. After washing twice in 1× Duolink wash buffer A for 5 min, cells were incubated with secondary antibodies conjugated with oligonucleotides (PLA probes anti-mouse MINUS and anti-rabbit PLUS) for 1 hour in a preheated humidity chamber at 37°C. See table S7 for details of antibodies. The samples were washed twice in Duolink washing buffer A for 5 min, and then the Duolink ligation solution was applied to the samples for 30 min in a preheated humidity chamber at 37°C. Cells were rinsed twice with Duolink washing buffer A for 5 min and the Duolink amplification-polymerase solution applied to the samples in a dark, preheated humidity chamber for 100 min at 37°C. The samples were then washed twice in 1× Duolink washing buffer B for 10 min at room temperature followed by a 1-min wash with 0.01× Duolink washing buffer B. The cells were stained with phalloidin for 1 hour in PBS with 2% donkey serum, rinsed three times with PBS, and incubated for 20 min in PBS/DAPI (NucBlue Live ReadyProbes Reagent, Invitrogen). Coverslips were mounted using ProLong Gold antifade reagent (Invitrogen), and confocal images were obtained using a Leica SP8 DMI8 confocal microscope. PLA signals were recognized as red fluorescent spots.

### Immunofluorescence of postmortem brains

To assess colocalization between GR aggregates and KIF5B in *C9*-ALS/FTD postmortem tissues, we made use of brain and spinal cord tissue previously collected in the UZ Leuven brain biobank in accordance with the ethics review board upon written informed consent. In this study, five *C9orf72* expansion carriers were used for the analysis in the frontal cortex of the brain and one *C9orf72* expansion carrier was used for the analysis in the spinal cord (for clinical information, see table S1). ALS patients without the *C9orf72* expansion were chosen as controls (table S1). Frozen tissue sections of 7 to 8 μm thickness were washed with Bond Wash Solution (Leica Biosystems) for 15 min at room temperature and stained overnight with the primary antibodies at 4°C (table S7). The following day, slides were washed three times with Bond Wash Solution before the secondary antibodies (table S7) were added for 90 min at room temperature. After three washing steps with Bond Wash Solution, the slices were mounted using ProLong Gold antifade reagent with DAPI (Invitrogen). Confocal images were obtained using a Leica SP8 DMI8 confocal microscope. Images were analyzed, formatted, and quantified with ImageJ software.

### Proteins and DPRs

Full-length human dynein with a SNAP_f_-tag on dynein heavy chain was expressed in *Spodoptera frugiperda* Sf9 cells and purified and labeled with fluorophores as described previously (*64*). A SNAP_f_ fusion of mouse BICD2N (not labeled with a fluorophore) was also produced from Sf9 cells using published methods (*64*). Native dynactin was purified from pig brain by SP-Sepharose–based purification as described (*64*).

Active kinesin-1 (1–560 truncation of KIF5B containing the motor domain and dimerization stalk) was purified from Rosetta 2 *Escherichia coli* (DE3, Novagen), transformed with the pET17-K560-GFP-His plasmid (*43*), and cultured at 37°C. Expression was induced with 0.1 mM isopropyl-β-d-thiogalactopyranoside (IPTG), with cells harvested 14 to 18 hours later by centrifugation for 20 min at 3000*g* (JLA 8.1 rotor) and resuspended in buffer A [50 mM Na-phosphate buffer (pH 8), 1 mM MgCl_2_, 250 mM NaCl, and 1 mM phenylmethylsulfonyl fluoride (PMSF)]. Following lysis by sonication, insoluble material was removed by centrifugation for 35 min at 27,000*g* (70 Ti rotor). Initial purification was carried out by Ni^2+^-affinity chromatography (HisTrap FF 5 ml, GE Healthcare) and elution with 50 mM Na-phosphate buffer, 1 mM MgCl_2_, 250 mM NaCl, 250 mM imidazole, 50 mM Mg-ATP, and 1 mM PMSF (pH 7.2). Protein was concentrated using Amicon 100-kDa 15-ml tubes at 4°C (4000*g* spin) and washed in buffer A. Further purification was achieved with a microtubule pull-down and release procedure, which enriches for the intact, active motor domain over degradation products (*65*). The protein sample was incubated with taxol-stabilized microtubules (15 mg ml^−1^) and 1 mM AMP-PNP (Sigma-Aldrich), and the mixture was overlaid on a glycerol cushion {BRB80 [80 mM Pipes (pH 6.9), 1 mM EGTA, 1 mM MgCl_2_], 20 μM taxol, and 60% glycerol (v/v)}. Centrifugation was performed with a TLA 100 rotor at 317,000*g* for 10 min. The pellet was resuspended in BRB80 with 50 mM KCl and 5 mM ATP and incubated for 10 min to trigger release of microtubule-bound motors. Centrifugation at 317,000*g* for 10 min yielded supernatant containing the released active motors, which were snap-frozen in small aliquots in 20% glycerol (v/v) and stored at −80°C.

Porcine tubulin conjugated to fluorophores or biotin was purchased from Cytoskeleton Inc. High-performance liquid chromatography–purified DPRs were purchased from Pepscan and were either labeled with a single Cy5 dye at the N terminus during synthesis or labeled non–site-specifically in-house using Alexa Fluor 488 Protein Labeling Kit (Thermo Fisher Scientific) using published methods (*66*).

### Single-molecule motility assays

Biotinylated and fluorescently labeled porcine microtubules stabilized with taxol and GMP-CPP (guanosine-5′-[(α,β)-methyleno]triphosphate), PEG (polyethylene glycol)–biotin passivated glass surfaces, and motility chambers were prepared as described previously (*64*). For assays with TMR-dynein, microtubules were polymerized in the presence of HiLyte488-tubulin, with the exception of the experiments documented in Figs. 5E and 6 (E to G) and fig. S20 (B and C) and in which HiLyte647-tubulin was used for conditions in the presence of Alexa Fluor 488–PR_30_. Tetramethyl rhodamine isothiocyanate (TRITC)–tubulin was used for KIF5B::GFP assays. Stabilized microtubules were immobilized on the biotinylated glass surface in the motility chamber for 30 s and subsequently washed once with motility buffer (30 mM Hepes, 5 mM MgSO_4_, 1 mM EGTA, 1 mM DTT). For this and subsequent steps, the pH of motility buffer was adjusted to 7.0 for KIF5B assays (kinesin motility buffer) and 7.3 for dynein assays (dynein motility buffer), as these conditions were optimal for movement of the respective motors.

KIF5B::GFP dimers (96 nM) were stored on ice before motility assays in kinesin motility buffer. For dynein assays, 138 nM TMR-dynein dimers, 585 nM dynactin, and 1.9 μM BICD2N dimers (molar ratio, 1:4:14) were mixed on ice for 5 to 10 min in dynein motility buffer. An independent assembly of dynein, dynactin, and BICD2N was used for each dynein assay chamber. KIF5B::GFP solutions were diluted 1:20 into kinesin motility buffer in the presence or absence of DPRs at the final concentrations indicated in Results. Dynein, dynactin, and BICD2N assembly mixtures were diluted 133-fold (with the exception of the complexes in Fig. 5C and fig. S21B, which were diluted 800-fold) in dynein motility buffer in the presence and absence of DPRs. Both final motility buffers also contained 25 mM KCl, α-casein (1 mg ml^−1^), 5 mM MgATP, and an oxygen scavenging system (1.25 μM glucose oxidase, 140 nM catalase, 71 mM 2-mercaptoethanol, and 25 mM glucose) to minimize photobleaching. Diluted protein samples were promptly injected into imaging chambers.

Imaging was performed at room temperature (23 ± 1°C) with a Nikon TIRF microscope system controlled with Micro-Manager and equipped with a 100× oil objective (Nikon APO TIRF, 1.49 NA), as well as with Coherent Sapphire 488 nm (150 mW), Coherent Sapphire 561 nm (150 mW), and Coherent CUBE 641 nm (100 mW) lasers. Images were acquired with an iXon^EM^+ DU-897E EMCCD (electron multiplying charged coupled device) camera (Andor), which gave pixel dimensions of 105 nm by 105 nm. Different channels were imaged sequentially through automated switching of emission filters [GFP, Cy3, and Cy5 (Chroma Technology Corp.)]. For each chamber, at least two dual-color acquisitions of 300 frames were made at the maximum achievable frame rate (~2 frames s^−1^) and 100-ms exposure per frame. Three chambers were imaged for every experimental condition with the exception of 1 μM PR30 in Fig. 5D and fig. S20A, where one chamber was imaged.

Kymographs were generated and manually analyzed using Fiji. Microtubule position was determined by the fluorescent tubulin signal or a projection of motor or DPR signal over the duration of the movie. From each chamber, typically 10 microtubules were selected for analysis across two movies (five microtubules per movie), with the exception of Fig. 5E and fig. S20 (B and C), where 15 microtubules were selected across three movies when assaying PR_30_ addition. Microtubules were chosen before visualizing the motile properties of associated motor complexes or pattern of DPR association. Preference was given to those microtubules that were relatively long and well separated from others in the field of view. Subsequent analysis of kymographs was carried out blind to the identity of the samples, with the exception of the experiments documented in Fig. 5 (D and E) and fig. S20. Blinding was not possible in these cases because either the magnitude of the effects on motility made it clear from which conditions the kymographs were derived or the batch of kymographs for analysis contained a single condition.

Interactions of fluorescently labeled motors with microtubules were operationally considered as binding events if they were ≥2 s (4 frames) in duration. As described previously (*64*), some processive complexes changed velocity within a run, which necessitated the calculation of velocities of individual constant-velocity segments. Run lengths were calculated from the total displacement of continuous movements of complexes, irrespective of changes in velocity. Pauses within or at the end of runs were scored only if the stationary event exceeded 1.5 s (≥3 frames). For Fig. 5C, background signal was quantified by generating kymographs from randomly selected microtubule-free regions of the coverslip of lengths equal to the median microtubule length of those used for analysis. Although kymographs displayed in Fig. 5A had background subtracted in Fiji with a rolling ball radius of 100 pixels for illustrative purposes, the raw data were used for all quantitative analysis.

### Quantification of peptide binding to microtubules in vitro

To quantify microtubule binding by Cy5-labeled DPRs to microtubules with or without C-terminal tubulin tails, TRITC-labeled microtubules were first polymerized as described above. Microtubules resuspended in BRB80 with 20 μM taxol were treated with a 1:50 (w/w) ratio of subtilisin (Sigma-Aldrich) to tubulin, or a buffer only control, for ~16 hours. Digestion was quenched with 5 mM PMSF and confirmed by gel electrophoresis and Coomassie staining. Microtubules were then pelleted, washed, and resuspended in fresh BRB80 with taxol before application to imaging chambers. Cy5-labeled DPRs in kinesin motility buffer were added to the chambers to a final concentration of 10 μM. For analysis, five microtubules were selected randomly from three images acquired from two independent chamber preparations. Mean Cy5 fluorescent intensity was then calculated using Fiji. Background signal was first subtracted with a rolling ball radius of 50 pixels to generate uniform fluorescent intensity across the region. Enrichment of fluorescence on microtubules was calculated by subtracting mean background intensity from microtubule-associated intensity. Background intensity was calculated from five lines per image equivalent to the median length of microtubules assessed, which were positioned in microtubule-free areas close to the selected microtubules.

### Analysis of motor pausing and detachment with PR_30_-decorated microtubules

Single-molecule motility data for KIF5B and dynein were acquired as described above. Additional analysis of the colocalization of pause and detachment sites for each motor with intense PR_30_ puncta was carried out in Fiji. Co-incidence was scored if there was at least a 2-pixel overlap between the PR_30_ signal and signal from the incoming motor complex. For the simulated control, randomized binding patterns of PR_30_ foci were generated by overlaying existing kymographs of motor trajectories with PR_30_ binding patterns from different microtubules or microtubule regions within the same chamber preparation. This procedure led to simulated PR_30_ banding patterns at an average density equivalent to the true patterns, which were used to account for the contribution of random chance to the frequency of colocalization events. Original and simulated images were assessed blind to their identities to avoid unconscious bias.

### Statistics

Statistical analyses and data plotting were performed using Prism 8. Normality of the data was tested using the D’Agostino & Pearson normality test. Data containing more than two groups showing normal distribution were analyzed with a one-way analysis of variance (ANOVA) test and Tukey post hoc test. Data that were not normally distributed were analyzed using the Kruskal-Wallis nonparametric test with Dunn’s correction for multiple comparisons. Data with two groups showing normal distribution were analyzed using Student’s *t* test; otherwise, the Mann-Whitney *U* test was used. A one-way ANOVA test with Dunnett’s correction was used for multiple comparisons to the control or Tukey’s correction when comparing between all conditions. A Fisher’s exact test was performed when comparing categorical variables. For the data in Fig. 1B, a linear mixed model with random effect of subject and of experiment was also applied and confirmed significance. *P < 0.05, **P < 0.01, ***P < 0.001, and ****P < 0.0001 were considered significant. Data values represent means ± SD, unless indicated otherwise.

## SUPPLEMENTARY MATERIALS

Supplementary material for this article is available at http://advances.sciencemag.org/cgi/content/full/7/15/eabg3013/DC1

## Appendix

View/request a protocol for this paper from *Bio-protocol*.
